# Molecular Epidemiology of Invasive Listeriosis due to *Listeria monocytogenes* in a Spanish Hospital over a Nine-Year Study Period, 2006–2014

**DOI:** 10.1155/2015/191409

**Published:** 2015-10-11

**Authors:** Jaime Ariza-Miguel, María Isabel Fernández-Natal, Francisco Soriano, Marta Hernández, Beatrix Stessl, David Rodríguez-Lázaro

**Affiliations:** ^1^Instituto Tecnológico Agrario de Castilla y León, Valladolid, Spain; ^2^Department of Clinical Microbiology, Complejo Asistencial Universitario de León, León, Spain; ^3^Institute of Biomedicine (IBIOMED), University of León, León, Spain; ^4^Public Health, School of Physiotherapy ONCE, Madrid, Spain; ^5^Institute of Milk Hygiene, Milk Technology and Food Science, Department for Farm Animals and Veterinary Public Health, University of Veterinary Medicine, Vienna, Austria; ^6^Microbiology Section, Department of Biotechnology and Food Science, Faculty of Sciences, University of Burgos, Burgos, Spain

## Abstract

We investigated the pathogenicity, invasiveness, and genetic relatedness of 17 clinical *Listeria monocytogenes* stains isolated over a period of nine years (2006–2014). All isolates were phenotypically characterised and growth patterns were determined. The antimicrobial susceptibility of *L. monocytogenes* isolates was determined in E-tests. Invasion assays were performed with epithelial HeLa cells. Finally, *L. monocytogenes* isolates were subtyped by PFGE and MLST. All isolates had similar phenotypic characteristics (*β*-haemolysis and lecithinase activity), and three types of growth curve were observed. Bacterial recovery rates after invasion assays ranged from 0.09% to 7.26% (1.62 ± 0.46). MLST identified 11 sequence types (STs), and 14 PFGE profiles were obtained, indicating a high degree of genetic diversity. Genetic studies unequivocally revealed the occurrence of one outbreak of listeriosis in humans that had not previously been reported. This outbreak occurred in October 2009 and affected three patients from neighbouring towns. In conclusion, the molecular epidemiological analysis clearly revealed a cluster (three human cases, all ST1) of not previously reported listeriosis cases in northwestern Spain. Our findings indicate that molecular subtyping, in combination with epidemiological case analysis, is essential and should be implemented in routine diagnosis, to improve the tracing of the sources of outbreaks.

## 1. Introduction

*Listeria monocytogenes* is an emerging foodborne pathogen capable of infecting animals and humans. It is the leading cause of death in reported cases of food poisoning [1]. The groups at highest risk of listeriosis are pregnant women, in whom this infection may cause late miscarriage or stillbirth, neonates, immunocompromised individuals, and the elderly, in whom it causes mostly septicaemia and meningoencephalitis [2, 3]. The incidence of listeriosis has recently been reported to be higher among the elderly than in other groups [4, 5]. The case-fatality rate is still increasing (20–30%) worldwide, despite antibiotic treatment [6, 7]. Noninvasive listeriosis is often associated with febrile gastroenteritis and sometimes with cutaneous forms, as observed in veterinary surgeons coming into direct contact with aborted foetuses from livestock [8, 9]. The disease is usually vertically transmitted during pregnancy or acquired by the consumption of contaminated food, particularly fresh and ready-to-eat products that are not heated before consumption [10, 11]. A European Food Safety Authority (EFSA) baseline study focusing on ready-to-eat (RTE) food indicated that smoked and marinated fish products carried the highest risk of* L. monocytogenes* contamination [1]. In the US, poultry meat was found to be responsible for most (63%) fatal cases of listeriosis [12]. A recent study showed that foodborne outbreaks in hospitalised patients on immunosuppression treatments were linked to hospital food (e.g., sandwiches and celery) [5].

*L*.* monocytogenes* consists of four discrete evolutionary lineages (I–IV) and 13 serotypes [13, 14]. Historically, diverse molecular typing methods, including PFGE, multilocus enzyme electrophoresis, and ribotyping, have been used to study the genetic diversity of the isolates involved in international outbreaks. Thus, epidemic clones (ECs) involved in geographically and temporally distant outbreaks or in large, single outbreaks have been defined [15]. In the last decade, ECs have been redefined on the basis of multi-virulence-locus sequence typing (MVLST), which is based on the analysis of six to eight genes [16]. Multilocus sequence typing (MLST), which is based on the analysis of seven housekeeping genes [17], has also been used for the definition of clonal groups. Sequence types (ST) are defined as a unique combination of MLST allele designations used in the MLST scheme, and clonal complexes (CC) are defined as groups of STs differing by only one housekeeping gene from other members of the group. An analysis of* L*.* monocytogenes* isolates from five continents by MLST demonstrated the existence of globally successful genetic groups [18]. Seven “epidemic clones” (ECs) have been defined by MVLST, each descended from a common ancestor with a similar temporal and spatial virulence profile [16, 17, 19, 20]. ECI, corresponding to the CC1 identified by MLST, and ECIV (CC2) appear to be cosmopolitan clones involved in many outbreaks [16].* L*.* monocytogenes* sequence type (ST) 6 (ECII) has been implicated in human meningitis with a fatal outcome [21].* L*.* monocytogenes* serogroups most frequently associated with clinical cases are serotype 4b, followed by 1/2b (genetic lineages I and III), and 1/2a (genetic lineage II) [14].* L*.* monocytogenes* serotype 1/2a is increasingly being isolated from cases of invasive listeriosis in Italy and Switzerland [21–24]. A link between isolates obtained from patients and isolates obtained from smoked fish has been reported in Scandinavian countries (Sweden, Norway, and Finland) and in eastern Spain [25–29]. Furthermore, actual outbreaks of listeriosis have been linked to* L*.* monocytogenes* serotype 1/2a and seem to be particularly prevalent in cheese processing plants [10, 30–33].

The incidence of listeriosis in Spain has increased steadily over the last decade. There were a reported 0.56 cases per 100,000 inhabitants from 2001 to 2007 [34]. Martínez et al. [35] reported 0.67 invasive listeriosis cases per 100,000 inhabitants in Valencia during the 2008–2010 period. In 2012, the notification rate for listeriosis cases in Spain was the second highest of any member state of the EU (0.93, versus an EU-wide rate of 0.41 per 100,000 inhabitants) [6].

Mortality rates are high for invasive listeriosis, justifying the use of combinations of molecular subtyping tools for the identification of clusters associated with outbreaks, tracing the source of the outbreak, and preventing further transmission. These methods were therefore combined in a retrospective study focusing on invasive listeriosis cases in León (2006–2014) and involving* in vitro* virulence testing.

## 2. Materials and Methods

### 2.1. Case Definition

All patients suffering from meningitis, bacteraemia, or infection during pregnancy were considered as potential cases of listeriosis. The infection was confirmed by the isolation of* L*.* monocytogenes* from a normally sterile site.

### 2.2. Description of the Hospital

The study has been carried out in the “*Complejo Asistencial Universitario de León*” (CAULE), a facility with about 800 beds located in the province of León in Northwest Spain. It serves an urban population of over 130,000 inhabitants and the total population of the metropolitan area has been estimated at over 490,000.

### 2.3. Clinical Cases

In total, there were 17 clinical cases of listeriosis at the CAULE from 2006 to 2014. These cases occurred in one premature newborn and 11 male and five female patients, aged from 31 to 89 years. In total, 11* L*.* monocytogenes* isolates were recovered from blood cultures, and six were recovered from cerebrospinal fluid (CSF). In some patients* L*.* monocytogenes* isolates were recovered from both blood cultures and CSF (*n* = 2) or from peritoneal fluid (PF; *n* = 1) or synovial joint fluid (JF; *n* = 1) (Table 1).

### 2.4. Isolation and Confirmation of* L. monocytogenes*

Clinical isolates of* L*.* monocytogenes* were streaked onto two selective chromogenic agar plates: ALOA (Agar Listeria Ottaviani & Agosti) medium (CHEMUNEX, Bruz Cedex, France) and RAPID L. mono agar (Bio-Rad Laboratories, Inc., Hercules, Ca, US). The bacteria were subjected to Gram staining and catalase and Christie Atkins Munch-Petersen (CAMP) tests. The collection strain* S. aureus* CECT 828 was used in the CAMP test, as recommended, to enhance* L. monocytogenes* haemolysis.

Each of the* L*.* monocytogenes* isolates was confirmed biochemically (API Coryne V.2.0; bioMérieux, Marcy l'Etoile, France) and by real-time PCR methods for differentiating between* Listeria* species [36–39]. The* L*.* innocua* CECT910,* L. monocytogenes* ITA1315, and* L*.* ivanovii* ATCC19119 reference strains served as positive controls in the PCR assays.

### 2.5. *L. monocytogenes* Growth Curves

Growth curves were determined by culture in brain heart infusion (BHI, Oxoid, Hampshire, UK), with the measurement of optical density at 600 nm in a Lambda 35 UV/VIS Spectrometer (PerkinElmer, Massachusetts, USA) over a 24-hour period of incubation at 37°C, with shaking at 180 rpm.

### 2.6. Antimicrobial Susceptibility Testing

The susceptibility of 17 clinical* L. monocytogenes* isolates to 16 antimicrobial agents was determined by E-tests on Mueller-Hinton agar supplemented with 5% sheep's blood, incubated under an atmosphere of ambient air at 35°C, with reading of the plates after 20–24 h. Susceptibility to antibiotics was interpreted applying the recommendations of the EUCAST for the antimicrobial susceptibility of* L. monocytogenes* [40]. The following antibiotics were tested: benzylpenicillin, ampicillin, imipenem, meropenem, erythromycin, clindamycin, gentamicin, vancomycin, daptomycin, linezolid, ciprofloxacin, moxifloxacin, tetracycline, tigecycline, rifampin, and cotrimoxazole.

### 2.7. Invasion Assays

We assessed the invasiveness of 17 clinical* L. monocytogenes* isolates in an epithelial HeLa cell culture assay, as previously described [41]. A well characterised clinical* L. monocytogenes* serovar 4b strain (P14) and its isogenic* prf*A gene deletion mutant (*Δprf*A) were included in the assay as controls. HeLa ATCC CCL-2 cells were maintained at 37°C, under an atmosphere containing 5% CO_2_. The invasiveness of each* L. monocytogenes* isolate was analysed in quadruplicate (2 independent invasion assays, with each isolate analysed in duplicate in each assay). Between passages 1 and 14, cell lines were maintained in Eagle's minimum essential medium (MEM; Gibco, San Diego, United States) supplemented with 2 mM L-glutamine, 10% foetal bovine serum, and 1% nonessential amino acids. Bacteria were resuspended in plain Eagle's minimum essential medium and used to infect HeLa cells at a multiplicity of infection (MOI) of 20 : 1, with the exception of the* Δprf*A mutant strain of* L. monocytogenes,* for which we used a MOI of 200 : 1.

### 2.8. Molecular Epidemiological Analysis

*L. monocytogenes* serogroups were defined according to a multiplex PCR targeting the specific target genes* lmo*0737,* lmo*1118,* ORF*2819,* ORF*2110, and* Listeria* spp.-specific* prs* published by Doumith et al. [42] and amended by Leclercq et al. [43] for PCR IVb-VI.* L*.* monocytogenes* clinical isolates were genomically characterised by pulsed-field gel electrophoresis (PFGE) with the restriction enzymes* Apa*I and* Asc*I, according to the standardised international protocol of PulseNet [44]. We analysed PFGE profiles with Bionumerics v.6.6 software (Applied-Maths NV, Sint-Martens-Latem, Belgium), to describe the genetic relationships between isolates. Dendrograms were constructed with the Dice similarity coefficient and the unweighted pair group mathematical average (UPGMA) clustering algorithm. Tolerance and optimisation values were set to 1.5%, in accordance with the recommendations of Martin et al. [45]. Simpson's index of diversity, which measures the probability of two unrelated strains sampled from the test population being placed in different typing groups [46], was calculated to compare the discriminative power of PFGE, via a website comparing partitions [47]. Fingerprints were interpreted according to the recommendations for foodborne pathogens [48]. Multilocus sequence typing (MLST) was performed as described by Ragon et al. [17]. Allele types were assigned for seven housekeeping loci,* abc*Z (ABC transporter),* bgl*A (beta glucosidase),* cat* (catalase),* dap*E (succinyl diaminopimelate desuccinylase),* dat* (D-amino acid aminotransferase),* ldh* (L-lactate dehydrogenase), and* lhk*A (histidine kinase), and the resulting sequence types (STs) were determined and compared, with the Institute Pasteur* Listeria monocytogenes* MLST database [49]. Sequence types (STs) were defined as a unique combination of MLST allele designations used in the MLST scheme, and clonal complexes (CC) were defined as groups of STs differing by only one housekeeping gene from other members of the group [17]. An allelic profile-based comparison, based on the use of a minimum spanning tree (MST) and the Pasteur Institute online tool, was performed to define the relationships between strains at the microevolutionary level.

### 2.9. Statistical Analysis

Statistical two-way analysis of variance (ANOVA) was used to evaluate differences in invasive capacity between isolates, based on the 95% confidence interval and Bonferroni multiple comparison tests to assess the differences in greater depth (GraphPad Prism v.5.0). The threshold *P* value for this test was set at 0.05.

## 3. Results

### 3.1. Patient Outcomes

The outcome was favourable after antibiotic treatment in 15 of the 17 patients. Progression was observed in case 4, a pregnant woman whose foetus died as a result of the infection. Patient 9 died after antibiotic treatment failure. Further information is provided in Table 1.

### 3.2. *L. monocytogenes* Isolation, Confirmation, and Growth Curves

**Figure 1:**
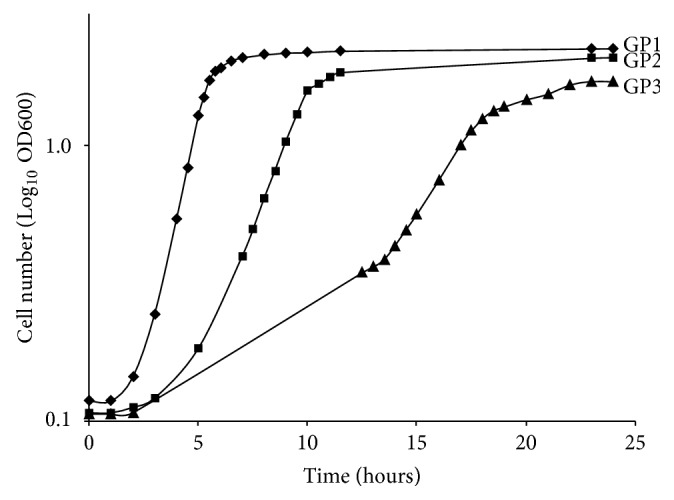
Growth patterns of 17 clinical isolates of* Listeria monocytogenes* causing invasive infections at the “Complejo Asistencial Universitario de León” from 2006 to 2014. Two of the isolates had growth patterns different from that of all the other isolates: isolate 6 (GP2) and isolate 7 (GP3), respectively.

All 17 clinical isolates were confirmed to be* L. monocytogenes* by phenotypic and genetic methods. *β*-haemolytic and lecithinase activities, at 24, 48, and 72 hours, were similar in all the clinical isolates. As expected, these activities were stronger in* L. ivanovii* ATCC19119, and* L. innocua* CECT910 displayed no activity. The isolates recovered from clinical cases 6 and 7 had specific growth patterns (GPs), reaching the exponential growth phase later than the other isolates (exponential growth phase from 5 to 12 hours and from 13 to 23 hours of incubation, resp.). The exponential phase in the other* L. monocytogenes* isolates began after about three hours and continued until about 6.5 hours of incubation. Representative growth curves for the isolates are shown in Figure 1.

### 3.3. Antimicrobial Susceptibility Testing

The antimicrobial susceptibility data for the 17 isolates tested are presented in Table 2. In the E-test method, all our isolates were found to be susceptible to benzylpenicillin, ampicillin, erythromycin, and cotrimoxazole. In 10 isolates, the MIC of meropenem was 0.38 to 0.75 mg/L. These isolates may be considered resistant, according to the strict criterion of the EUCAST susceptibility breakpoint. No susceptibility breakpoints have been identified for the other 11 antibiotics by EUCAST, and the MICs of these antibiotics ranged between 0.125 and 4 mg/L.

### 3.4. Invasion Assays

**Figure 2:**
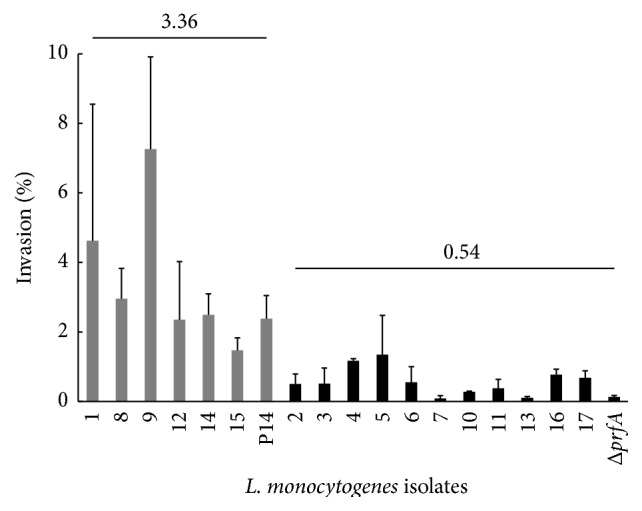
Invasion assays for the 17 clinical isolates of* Listeria monocytogenes* in HeLa epithelial cells. The mean number of internalised bacteria as a percentage of the initial inoculum is shown on the *y*-axis. The error bars show the standard error of two independent experiments, each performed in duplicate. The wild-type* L. monocytogenes* P14 and noninvasive* L. monocytogenes ΔprfA* strains were included, to assess the reproducibility of the experiments.

All clinical isolates of* L*.* monocytogenes* were tested in invasion assays with HeLa epithelial cells. The actual mean MOI used for cell infection with the clinical isolates was 17 : 1 (standard error: 0.83). The recovery rates for clinical isolates ranged from 0.09% to 7.26% (median: 1.62, standard error: 0.46). As expected, the recovery rate for the noninvasive* Δprf*A mutant strain was very low (0.13%, standard error: 0.04). Invasiveness was between 0.04 and 3.1 times higher than that of the* L. monocytogenes* serovar 4b clinical control strain (P14) (Figure 2). The isolates clustered into two significantly different groups: those with a high invasion rate similar to that of the wild-type strain P14 (3.36 ± 0.74) (isolates from clinical cases 1, 8, 9, 12, 14, and 15) and isolates with a low invasion rate, similar to that of the isotypic* Δprf*A strain (0.54 ± 0.12) (isolates from clinical cases 2, 3, 4, 5, 6, 7, 10, 11, 13, 16, and 17 (Figure 2)).

### 3.5. Molecular Epidemiological Analysis

**Figure 3:**
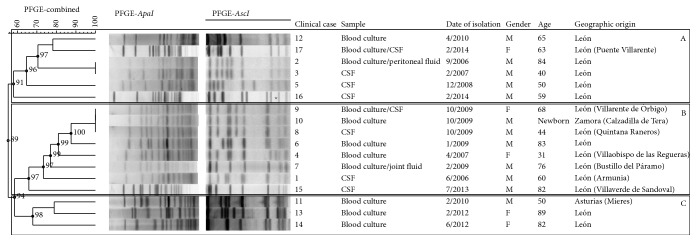
Genetic relationships between 17 clinical isolates of* Listeria monocytogenes*, based upon comparison of pulsed-field gel electrophoresis profiles obtained with the restriction enzymes* Apa*I and* Asc*I. The dendrogram was produced with a Dice similarity coefficient matrix, using the unweighted pair group method with arithmetic mean (UPGMA). Tolerance and optimisation values were set to 1.5%. Clusters are arbitrarily designated A to C. Scale bar indicates similarity values.

Genetic characterisation by PFGE with the restriction enzyme* Apa*I revealed 13 different pulsotypes, and characterisation with* Asc*I discriminated between 14 genotypes (Simpson's index of diversity values of 0.949 and 0.971, resp.). Fingerprinting revealed the presence of 8–20 DNA fragments between about 40 and 560 kb in size. PFGE analyses combining the results obtained with both restriction enzymes identified 14 unique pulsotypes, resulting in a Simpson's index of diversity of 0.971. The clinical isolates displayed 55% similarity and formed three clusters, arbitrarily designated A to C. Cluster A contained six isolates recovered from 2006 to 2014 (58% similarity). Cluster B contained eight isolates recovered from 2006 to 2013 (66% similarity). Cluster C consisted of three isolates (68% similarity) collected from 2010 to 2012. Interestingly, the isolates from clinical cases 8, 9, and 10 on one hand and those from cases 2 and 3 on the other hand had indistinguishable pulsotypes (pulsotypes D and I, resp.). The genetic relationships between* L*.* monocytogenes* isolates, based on the combined PFGE-genetic profiles obtained with the restriction enzymes* Apa*I and* Asc*I, are shown in Figure 3.

**Figure 4:**
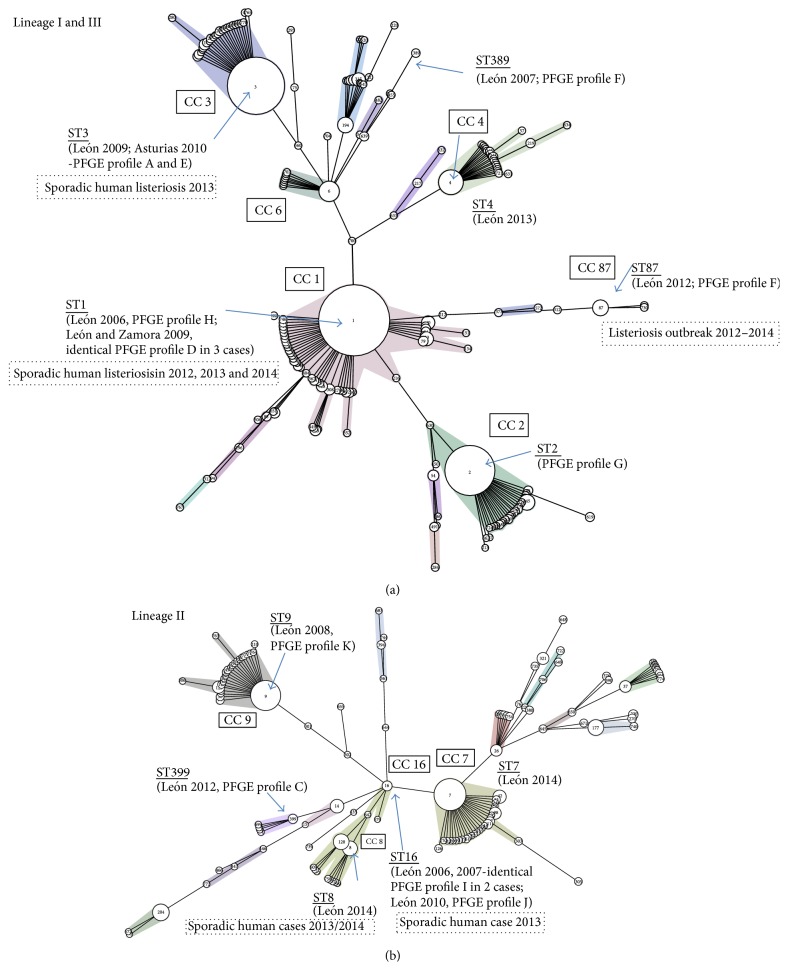
Multilocus sequence typing of 17* Listeria monocytogenes* isolates from sporadic cases of human listeriosis in Spain during the 2006–2014 period. The sequence types were clustered according to the sequence of the* abc*z housekeeping gene, using a minimum spanning tree (MST) tool available from the Pasteur Institute MLST database (http://www.pasteur.fr/recherche/genopole/PF8/mlst/). The STs from genetic lineages I and III (a) and genetic lineage II (b) found in this study are underlined. Sporadic listeriosis cases and outbreaks in Spain (2012–2014) listed in the Pasteur Institute MLST database are shown in boxes outlined with dotted lines.* L*.* monocytogenes* sample origins and PFGE profiles are included in each MST. The coloured zones surrounding groups of STs indicate clonal complexes (CC) differing by only one gene from other members of the group.

MLST analysis of 17* L*.* monocytogenes* isolates from clinical cases of listeriosis identified 11 STs (Simpson's index of diversity: 0.926). The oldest and globally most prevalent epidemic clones (ST1, ST2, ST3, ST4, ST7, ST8, and ST9) were represented among the STs of the clinical cases observed in the Spanish regions of León, Asturias, and Zamora in 2006–2014 (Table 1; Figures 4(a) and 4(b)). Ten isolates (58.8%) were assigned to genetic lineage I or III. Four of these isolates were ST1 isolates (3 with PFGE profile D and 1 with PFGE profile H) recovered from human patients from the neighbouring provinces of León and Zamora during 2006 and 2009. ST3 isolates were obtained from two patients from the provinces of León and Asturias in 2009 and 2010, respectively. These cases were not related and the isolates concerned had different PGFE profiles (A and E). Furthermore, ST2, ST4, ST87, and ST389 were sporadically observed, in one isolate each. The* L*.* monocytogenes* isolates from genetic lineages II (*n* = 7; 41.2%) were more evenly distributed in the MST. The most common allelic profile was ST16 (*n* = 3; 17.6%). ST7, ST8, ST9, and ST399 were observed sporadically, in one isolate each (Table 1).

## 4. Discussion

Most of the reported cases of listeriosis occur in high-income countries, this infection being largely underreported in developing countries. Hospitalisation records show listeriosis to be the third most costly zoonotic disease in the US [34, 50]. Patients often suffer from comorbid diseases and are immunocompromised, and long-term antibiotic treatment with ampicillin, amoxicillin, and gentamicin may be required. Some* L. monocytogenes* strains can survive treatment with cephalosporin or erythromycin [51]. Livestock and processed foods seem to serve as a source of antibiotic resistance. Some authors have reported increases in the frequency of multidrug-resistant strains (e.g., resistant to amoxicillin-clavulanate and chloramphenicol) [25, 52]. No resistance to benzylpenicillin, ampicillin, erythromycin, or cotrimoxazole was found in our isolates. Remarkably, 10 isolates were classified as resistant to meropenem according to the EUCAST breakpoint for this antibiotic, potentially discouraging its use to treat meningitis. All patients were treated with betalactams (ampicillin or amoxicillin), mostly in combination with gentamicin. Treatment outcome was favourable in 15 patients (88.2%). Mortality is known to be high in patients with invasive listeriosis, severe underlying diseases, meningoencephalitis, and inadequate antimicrobial treatment. The early administration of antibiotics, such as ampicillin or cotrimoxazole, which have rapid bactericidal activity against the pathogen, is essential for cure.

The 17* L*.* monocytogenes* clinical isolates included in this study had phenotypic properties consistent with full virulence: *β*-haemolysis mediated by the product of the listeriolysin gene (*hly*) and lecithinase activity due to the presence of phospholipases (*plcA* and* plcB*). Some differences in growth patterns and invasiveness were observed. Growth pattern (GP) 1 was observed in all but two of the* L*.* monocytogenes* isolates (87.5% of all isolates). The* L*.* monocytogenes* isolates from cases 6 and 7 (STs 3 and 2) reached the exponential growth phase later than the other isolates (Figure 1). In cell culture assays, invasiveness varied considerably between clinical isolates, with recovery rates ranging from 0.09% to 7.26% (mean 1.62%, standard error 0.46) (Figure 2). The recovery rates for five clinical isolates were reported to range from 4.3% to 30% in a previous study on Vero cells [53].* L*.* monocytogenes* isolates 9, 1, and 8 (all ST1) had the highest levels of virulence in cell culture assays* in vitro*, as shown by their invasiveness in HeLa cells (invasiveness up to three times that of the wild-type P14 strain). This finding is consistent with those of most previous studies, reporting that lineage I (4b) strains seem to be more virulent and better able to withstand the adverse conditions present in the stomach of the host [19]. ANOVA revealed the existence of two groups of isolates. Group I consisted of six clinical isolates (35.3%) and the wild-type P14 strain, all with significant levels of invasiveness. By contrast, group II contained 11 isolates (64.7%) with nonsignificant levels of invasiveness, similar to that of the isogenic control strain* Δprf*A.

In our study of invasive listeriosis cases, the percentage of lineages I and III isolates of* L*.* monocytogenes* was similar to that of lineage II isolates (58.8% versus 41.2%, resp.). The isolates responsible for the death of a 68-year-old woman and a foetus were assigned to ST1 and ST389, respectively (lineage I/III, clinical cases 9 and 4). Overall, PFGE identified 14 genotypes and MLST identified 11 genotypes among the 17* L*.* monocytogenes* clinical isolates, yielding values of Simpson's index of diversity of 0.971 and 0.926, respectively. This indicates a high level of genomic diversity among the clinical isolates (only 55% similarity on PFGE) despite their recovery at the same hospital, consistent with the findings of previous molecular epidemiology studies.

An analysis of the genomic relationships between isolates unequivocally revealed the occurrence of one previously unreported outbreak of listeriosis in humans. This outbreak occurred in October 2009 and affected three patients living in neighbouring towns (clinical cases 8, 9, and 10: pulsotype D, ST1). Isolate 10 had a significantly lower invasion capacity than isolates 8 and 9 (Figure 2), but this difference may simply reflect the method used, with invasion capacity being evaluated* in vitro*.* L*.* monocytogenes* ST1 isolates from Spanish patients are also present in the Pasteur Institute MLST database (2012–2014; Figure 4(a)). Moreover, an epidemiological connection was identified between two isolates from 2006 and 2007, both of which belonged to ST16 and had a PFGE profile I. Data for* L*.* monocytogenes* ST16 and ST8 isolates from sporadic clinical cases of human listeriosis are also available from the MLST database (Figure 4(b)). Many previous molecular epidemiology studies have detected otherwise unreported outbreaks of listeriosis. In a recent Spanish study, a large proportion of the clinical isolates had indistinguishable pulsotypes, suggesting the possible occurrence of listeriosis outbreaks related to international foodborne outbreaks. Most of these isolates were assigned to ECI (4b; CC1; 46.2%) and ECIII (1/2a; CC11; 33.3%) [54]. Thus, molecular epidemiology studies of* L*.* monocytogenes* can help to identify and trace the sources of outbreaks that would otherwise pass unnoticed.

Interestingly, this is the second time that ST87 has been linked to human disease (it was isolated in León in February 2012). Pérez-Trallero et al. [55] recently reported the occurrence of two outbreaks affecting 15 people and caused by ST87 strains in Guipúzcoa (northern Spain) in 2013 and 2014. Our study demonstrates that ST87 was already circulating in the Spanish clinical environment before the outbreak in Northern Spain. It would be interesting to follow the dissemination of this clone to assess its potential emergence.

In conclusion, this retrospective study focused on invasive* L*.* monocytogenes* infections in a Spanish healthcare institution over a nine-year study period. Molecular epidemiology studies clearly revealed the occurrence of a previously unreported outbreak of listeriosis in Northwest Spain. Our findings, along with those of previous studies [54–57], indicate that molecular epidemiology studies can help to identify and trace the source of the outbreaks that might otherwise pass unnoticed. Better centralised collection and subtyping of clinical isolates of* L*.* monocytogenes* would improve listeriosis monitoring, making it possible to trace the sources of Spanish outbreaks and to prevent cross-border outbreaks.

## Figures and Tables

**Table 1:** Clinical cases of invasive *Listeria monocytogenes* infections in a Spanish hospital over a nine-year study period, 2006–2014.

Sample	Sex	Age	Date	Source	Clinical diagnosis	Antibiotic treatment	Province	PFGE pulsotype	MLST ST
Genetic lineages I and III									
1	M	60	Jun. 06	CSF	Meningoencephalitis	Ampicillin	León	H	1
8	M	44	Oct. 09	CSF	Meningoencephalitis	Ampicillin/gentamicin	León	D	1
9^†^	F	68	Oct. 09	Blood culture/CSF	Meningoencephalitis/sepsis	Ampicillin/gentamicin/vancomycin	León	D	1
10	M	NB	Oct. 09	Blood culture	Sepsis	Ampicillin/gentamicin	Zamora	D	1
7	M	76	Feb. 09	Blood culture/JF	Arthritis/bacteraemia	Ampicillin/gentamicin	León	G	2
6	M	83	Jan. 09	Blood culture	Bacteraemia	Ampicillin/gentamicin	León	E	3
11	M	50	Feb. 10	Blood culture	Sepsis	Ampicillin/gentamicin	Asturias	A	3
15	M	82	Jul. 13	CSF	Meningoencephalitis	Ampicillin/gentamicin	León	L	4
13	F	89	Feb. 12	Blood culture	Sepsis	Ampicillin/gentamicin	León	B	87
4^†*∗*^	F	31	Apr. 07	Blood culture	Fever in pregnant woman	Amoxicillin clavulanate	León	F	389

Genetic lineage II									
5	M	50	Dec. 08	CSF	Meningoencephalitis	Ampicillin/gentamicin	León	K	9
2	M	84	Sep. 06	Blood culture/PF	Peritonitis/sepsis	Ampicillin/vancomycin	León	I	16
3	M	40	Feb. 07	CSF	Meningoencephalitis	Ampicillin/gentamicin	León	I	16
12	M	65	Apr. 10	Blood culture	Sepsis	Ampicillin/gentamicin	León	J	16
14	F	82	Jun. 12	Blood culture	Sepsis	Ampicillin/gentamicin	León	C	399
16	M	59	Feb. 14	CSF	Meningoencephalitis	Ampicillin/gentamicin	León	M	7
17	F	63	Feb. 14	Blood culture/CSF	Meningoencephalitis/sepsis	Ampicillin/gentamicin	León	N	8

^*∗*^Foetal death. Full recovery of the mother; ^†^Deceased.

PFGE, pulsed-field gel electrophoresis; MLST, multilocus sequence typing; NB, premature newborn; CSF, cerebrospinal fluid: JF, joint fluid; PF, peritoneal fluid.

**Table 2:** Antimicrobial susceptibility of 17 *L. monocytogenes* isolates in the E-test.

Antibiotic	MIC (mg/L)			Susceptibility breakpoint (mg/L)^a^	% susceptibility
	Range	50%	90%		
Benzylpenicillin	0.064–1	0.25	0.75	≤1	100
Ampicillin	0.064–1	0.25	0.75	≤1	100
Imipenem	0.125–0.19	0.19	0.19	—	—
Meropenem	0.19–0.75	0.38	0.38	≤0.25	58.8
Erythromycin	0.125–0.38	0.25	0.38	≤1	100
Clindamycin	0.25–8	2	4	—	—
Gentamicin	0.094–1	0.25	0.5	—	—
Vancomycin	0.75–1.5	1.5	1.5	—	—
Daptomycin	0.75–1.5	1	1.5	—	—
Linezolid	1-2	2	2	—	—
Ciprofloxacin	0.5–2	1	1.5	—	—
Moxifloxacin	0.19–0.5	0.38	0.5	—	—
Tetracycline	0.094–1.5	1	1.5	—	—
Tigecycline	0.094–1.5	0.125	0.25	—	—
Rifampin	0.023–0.19	0.094	0.125	—	—
Cotrimoxazole	0.008–0.023	0.012	0.019	≤0.06	100

^a^According to EUCAST antimicrobial susceptibility breakpoints for *L. monocytogenes*.

## Abbreviations

CAULE: Complejo Asistencial Universitario de León
BHI: Brain heart infusion
MOI: Multiplicity of infection
MLST: Multilocus sequence typing
ST: Sequence type
MST: Minimum spanning tree
CC: Clonal complex
PFGE: Pulsed-field gel electrophoresis
GP: Growth pattern.
